# Stress and Strain: Differentiating the Responses to High and Moderate Heat Loads and Subsequent Recovery in Grain-Fed Feedlot Steers—Metabolic Hormones

**DOI:** 10.3390/ani15020251

**Published:** 2025-01-17

**Authors:** Gene Wijffels, Megan L. Sullivan, Sally Stockwell, Suzie Briscoe, Roger Pearson, Stephen T. Anderson, Yutao Li, Cintia C. de Melo Costa, Russell McCulloch, John B. Gaughan

**Affiliations:** 1 CSIRO Agriculture and Food, Queensland Bioscience Precinct, St Lucia, QLD 4067, Australia; sally.stockwell@csiro.au (S.S.); suzie.briscoe@csiro.au (S.B.); yutao.li@csiro.au (Y.L.); russell.mcculloch@csiro.au (R.M.); 2 School of Agriculture and Food Sustainability, The University of Queensland, Gatton, QLD 4343, Australiaj.gaughan@uq.edu.au (J.B.G.); 3 School of Biomedical Sciences, The University of Queensland, St Lucia, QLD 4067, Australia; stephen.anderson@uq.edu.au; 4 Faculty of Agricultural and Veterinary Sciences, São Paulo State University (Unesp), Via de Acesso Paul Donato Castellane s/n, Jaboticabal 14884-900, SP, Brazil

**Keywords:** feedlot cattle, hyperthermia, allostasis, thyroid hormones, leptin, adiponectin, prolactin, TSH

## Abstract

The extent of endocrine changes in response to various levels of heat stress and subsequent recovery is not well understood. Two cohorts of 12 Black Angus steers were housed in climate-controlled rooms (CCR) and subjected to three thermal periods: PreChallenge (5 d), Challenge (7 d) and Recovery (5 d). PreChallenge and Recovery provided thermoneutral conditions. The Challenge simulated a strong heatwave. Finally, the steers were maintained in outdoor pens for 38 d. Rumen temperature (RumT), respiration rate (RR) and panting score (PS) were intensively measured in the CCR. Dry matter intake (DMI) was determined daily. The steers were bled most days, and a rectal temperature (RecT) was taken also. Plasma concentrations of TSH, prolactin, T3, T4, insulin, leptin and adiponectin were determined. During the Challenge, RumT, RecT, RR and PS rose; DMI was reduced. Plasma T3, T4 and adiponectin levels fell also. In Recovery, RumT, RecT, RR and PS fell below the PreChallenge mean. DMI partially recovered. T4 and adiponectin levels remained suppressed alongside lowered insulin. There were linear relationships between T3 concentration and THI, and T3 and T4 levels and DMI only. We highlight comparisons with previously reported metabolic hormone responses of grain-fed Black Angus steers to a moderate-heat-load challenge.

## Simple Summary

Rising summer temperature averages along with greater frequency, intensity and duration of moderate and severe heatwaves are negatively impacting livestock welfare and productivity. We are investigating the immediate and post-heatwave effects (strain) of different levels of heat stress on grain-fed feedlot steers. Under a moderate heat load challenge, the steers adjust by reducing feed intake and increasing drinking and sweating. In recovery, they soon return to normal. However, the high heat load challenge appears to push the steers into a new less productive physiological state in recovery and sometime after. This study follows the changes in the major metabolic hormones during and after a high heat load challenge. The findings reinforce the idea that steers are altered by high heat load, and this new state directs them to reduced feed intake and slower growth, which ensures a lower metabolic rate, and less heat production by the body. In this state, they are much less vulnerable to the next severe heatwave.

## 1. Introduction

The immediate impacts of heat stress on livestock, besides rising core temperature, are elevated water consumption, respiration rates and evaporative water loss, alongside reduced dry matter intake (DMI) [1,2]. Rising temperatures due to climate change will negatively impact global beef productivity. Based on the reduction in DMI alone, Thornton et al. (2022) [3] have estimated losses of USD 11.4 billion in global beef production by 2045 under the most benign Intergovernmental Panel on Climate Change GHG emission scenario [4]. Fittingly, over recent decades, there have been concerted efforts by the major beef cattle production industries, especially in the feedlot sector, to develop appropriate measures or indices of heat stress or heat load [5,6,7] and devise mitigation strategies using infrastructure (shade and sprinklers) and animal management (e.g., ration changes) [8,9,10,11,12].

However, the heat load intensity (level of stress) and duration can result in profoundly different outcomes (strain) for the animal and the production system. In our recent paper, we examined the major physiological and performance responses of grain-fed Black Angus steers to a high heat load (HHL) in detail [13]. For this experiment, 600 kg steers were subjected to a simulated severe heatwave which commenced with a sudden rise in maximum air temperature to 38–41 °C for 3 days. In tracking their physiological responses during Challenge, Recovery and PENS, we concluded that the steers underwent an allostatic response. Allostasis can be described as an altered state that may result in new set points and constraints or postpone normal function such as growth and/or reproduction [14,15]. The main finding is that the steers did not or could not return to the PreChallenge state in Recovery. In contrast, a homeorhetic response was discerned in the moderate-heat-load (MHL) challenge experiment [16]. These steers enlisted appropriate physiological adjustments as they moved from thermocomfort to heat stress and thermocomfort in Recovery. The MHL experiment used the same facility and was of similar experimental design: PreChallenge in thermoneutral conditions (4 d), Challenge (7 d) with a diurnal temperature range of 28–35 °C (a diurnal THI range of 73–84), returning to thermoneutral conditions in Recovery (7 d).

In the current study, we followed the responses of the metabolic hormones (via the plasma concentrations) of the steers in the HHL experiment. The main hypothesis was that the metabolic hormone responses in the HHL experiment would differ from those of the MHL experiment [17] as other mechanisms at the physiological, organ and cell levels are recruited to ensure survival. As a background, the dominant feature of the endocrine milieu of the MHL experiment was the substantially and consistently reduced plasma leptin concentrations; it was also characterised by period-specific fluctuations in T4, insulin and adiponectin [17]. The conclusion was that during the thermal challenge, the endocrine milieu allowed the MHL steers to conserve energy and constrain anabolism. In Recovery and PENS, they transitioned rapidly to compensatory growth so that by the end of the interval in outdoor pens, they were not different from feed-restricted thermoneutral (FRTN) counterparts [17].

The literature is replete with studies of the responses of various metabolic hormones to heat stress in ruminants. While some reports show similarities, others differ markedly and are occasionally contradictory with other reports. Life stage, age, diet or ration, previous experience, and housing all influence hormone responses during and after heat stress. However, the varied responses also direct us to the question of the level of stress and the strain that is incurred.

## 2. Materials and Methods

### 2.1. Animal Experiment

A detailed description of animal husbandry, diet, housing, climatic regime and analyses of animal performance and physiological responses can be found in [13]. The study was conducted from January–May 2017.

In brief, on arrival at the Queensland Animal Science Precinct (Gatton, Queensland, Australia), 24 Black Angus yearling steers undertook a feedlot induction vaccination regime and parasite treatment while on pasture (see [16] for details). The steers were allocated to two cohorts (1 and 2) of 12 animals balanced for body weight and temperament. Cohort 2 remained on pasture for 3 additional weeks, and Cohort 1 proceeded through the next two phases, feedlotting and the thermal challenge experiment in the climate-controlled rooms (CCR). Just before entry into the feedlot phase (60 d), each animal was administered with a hormonal growth promotant implant (Synovex^®^, Zoetis Australia, Sydney, Australia) and a rumen temperature bolus (SmartStock, Pawnee, OK, USA). The cohort was kept in outdoor feedlot pens for the 60 d, over which time they were ‘stepped up’ from a starter feedlot ration to a finisher ration [13]. Feedlot entry body weight (mean ± SD) was 493 ± 33.5 kg (n = 24).

Cohorts 1 and 2 entered the CCR on 8 March 2017 and 28 March 2017, respectively. Thus, these steers had experienced typical summer conditions in outdoor pens in the weeks before entry to the CCR. Cohort 1 in the 28 d before CCR entry experienced mean daily minimum and maximum air temperatures (±SD) of 20.9 ± 1.7 and 33.2 ± 3.6 °C, respectively. The corresponding mean daily minimum and maximum THIs (±SEM) were 68.6 ± 2.9 and 80.7 ± 2.9. Cohort 2 entered the CCR 20 d after the Cohort 1 entry. In the 28 d before CCR entry, Cohort 2 experienced mean daily minimum and maximum air temperatures (TA) of 20.2 ± 1.7 and 30.7 ± 2.5 °C, respectively, and the mean respective mean daily minimum and maximum THIs were 67.6 ± 2.8 and 78.9 ± 2.1. The mean body weight (±SD) 4–5 days before entry to the CCR was 603.4 ± 38.6 kg, and the mean DMI in the two days before CCR entry was 13.3 ± 1.6 kg/head/day.

**Figure 1:**
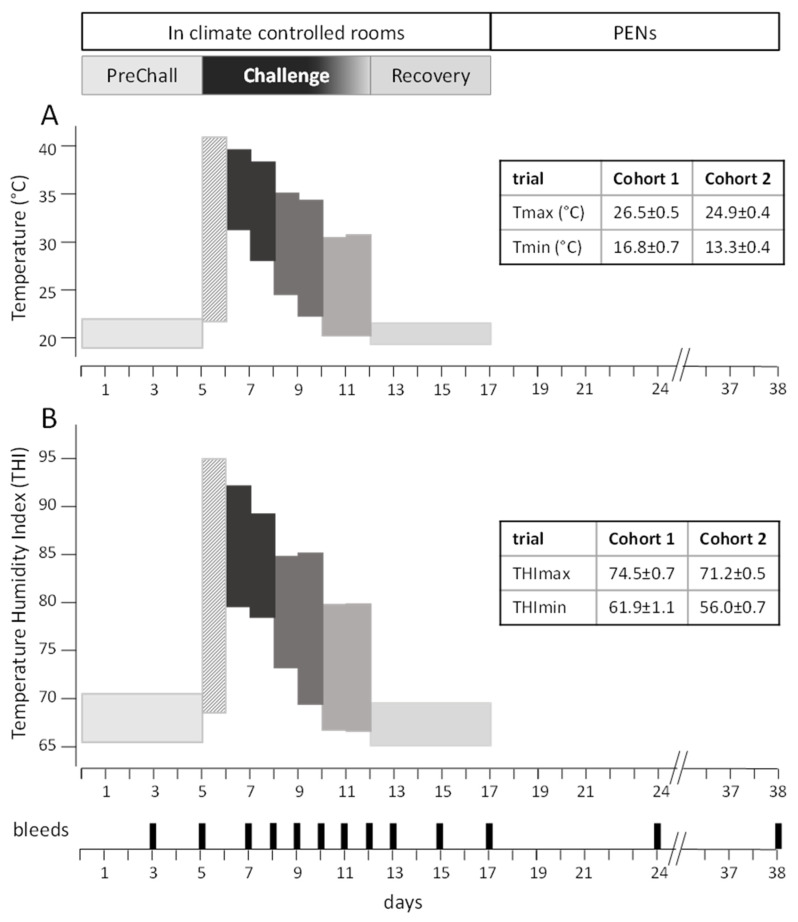
The climatic conditions experienced by two consecutive cohorts of 12 steers (Cohorts 1 and 2) while in climate-controlled rooms (CCRs) and outdoor feedlot pens. For the 17 days in the CCR, the steers were subjected to three sequential climatically different periods: PreChallenge (PreChall, 5 d), Challenge (7 d) and Recovery (5 d). The diurnal ranges of air temperature (**A**) and Temperature Humidity Index (THI, panel (**B**)) for each day are shown. For the final period, PENS, the steers were retained in feedlot pens for 20 days and exposed to autumnal conditions. The mean (±SEM) daily maximum and minimum air temperatures (Tmax and Tmin) and mean (±SEM) daily maximum and minimum THI (THImax and THImin) experienced by Cohorts 1 and 2 while in PENS are given in the inserted tables.

All 12 animals of each cohort were housed in individual pens (2.5 m × 2.5 m) in the CCR for 17 d and subsequently returned to the outdoor feedlot pens for 20 d (PENS). The steers had ad lib access to feed (finisher diet) and water throughout. The facility and housing are described in detail in [16]. The climatic regime delivered in the CCR is presented in Figure 1. The regime consisted of three sequential climatic periods: PreChallenge, Challenge and Recovery. Thermoneutral conditions were maintained in PreChallenge (d 1–5) and Recovery (d 13–17) with minimal diurnal variation in TA and THI. In PreChallenge, the mean (±SEM) daily TA was 20.7 ± 0.27 °C, and the mean daily THI was 67.8 ± 0.29. For the Recovery period, the mean daily TA was 20.4 ± 0.12 °C, and the mean daily THI was 67.3 ± 0.18.

The 7 d Challenge period (d 6–12 in the CCR) was designed to mimic a strong heatwave that occurred in early January 2014 in the southeast Queensland cattle finishing region [13]. This historical heatwave was characterised by a sudden large increase in high daytime TA and incremental falls in TA in two-day steps over the following days. In following this pattern during the Challenge period, the first day (d 6 in the CCR) delivered a rapid rise in daytime TA from a preceding cool evening i.e., a transition from a minimum TA of 21.7 °C (on average) to a maximum TA of 40.9 °C (on average) (Figure 1A).

The following formula [18] was applied to calculate the THI:

THI = (0.8 × TA) + {[(RH/100) × (TA − 14.3)] + 46.3}.

The minimum and maximum THI for d 6 were 68.5 and 94.5, respectively (Figure 1B). The following 2 days maintained both high daytime maximum TA (38.3–39.5 °C) and high night-time (minimum) TA (28.1–28.7 °C). The minimum THIs ranged between 78.6 and 79.6, and maximum THIs ranged between 89.1 and 92.1 (Figure 1B). The maximum TA was reduced on d 9 and 10 to 34.3–34.9 °C, with the night-time (minimum) TA lowered to 24.7 and 22.3 °C. The corresponding maximum and minimum THIs were 84.8–85.1 and 69.4–73.1 (Figure 1B). On d 11 and 12, daily maximum and minimum TAs were further lowered to 30.4–30.7 °C, and 20.1–20.3 °C, respectively (see Figure 1A). The daily minimum and maximum THIs for d 11 and 12 were approximately 66.6 and 80.2 (Figure 1B). Under these conditions, the daily maximum THIs for the first five days of the Challenge (d 6–10) were >84 and thus were in the Emergency THI category [19]. Days 11 and 12 fall into the Danger category (THI 79–83). The conditions in PreChallenge and Challenge fell into the Normal category (THI < 74).

On d 18, all animals were returned to outdoor feedlot pens where they experienced ‘autumn’ or ‘fall’ climatic conditions with mean maximum daily TA in the mid-20 °C range, and daily minima ranging between 10 and 20 °C (see Figure 1A). Mean daily THI maxima were in the Normal THI category (Figure 1B).

### 2.2. Physiological Measures

For the 17 d in the CCR, respiration rate (RR), panting score (PS) and water consumption were recorded by trained staff at two-hourly intervals in PreChallenge and Recovery during working hours, and hourly during Challenge over 24 h. Rectal temperature (RecT) was taken at 0700 h daily on d3, 5, 7–13, 15 and 17. Individual DMI was taken in the CCR, but only at the pen level during PENS. The methodology and housing are given in much detail in [16]. Body weights were not recorded during the CCR phase due to equipment failure. The RumT bolus data were radio-transmitted at 10 min intervals throughout to allow near-real-time monitoring of individual animal RumT. The data were transferred to a database for later analyses.

### 2.3. Plasma Hormone Assays

Blood samples were collected from each animal by jugular venepuncture into 10 mL EDTA vacutainer tubes at 0730. The bleed schedule is presented in Figure 1. Plasma was harvested by standard methods, placed on dry ice for transport and stored at −80 °C until required. Concentrations of plasma prolactin, TSH, adiponectin and leptin were determined using sandwich enzyme-linked immunosorbent assays (ELISAs), and measurement of the plasma thyroxine (T4) concentrations was performed by a competitive binding ELISA. Plasma insulin levels were determined by radio-immunoassay (TKIN2, Coat-a-Count Insulin, Siemens Healthcare Diagnostics, Los Angeles, CA, USA). The details for each assay method were previously described [17]. All samples were assayed in triplicate, and standards in quadruplicate. The performances of the ELISAs are given in Supplementary Table S1.

The plasma triiodothyronine T3 concentrations were determined by a competitive binding ELISA also. A monoclonal antibody to T3 (Abcam, [3A6] Cat. Number Ab1981) was diluted to 0.2 µg/mL in a carbonate coating buffer (0.1 M Na_2_CO_3_ pH 9.6). Clear 384-well plates (Perkin Elmer, Hopkinton, MA, USA) were coated overnight at 4 °C with the antibody solution (50 µL/well). After washing, plates were blocked in 2% skim milk powder in TBST for 30 min at RT. T3-BSA (SQX-CBS-9010, Squarix, Marl, Germany) was prepared as a standard in a 200 µM solution of 8-anilino-1-naphthalene sulphonic acid (ANSA) in BSA/TBST and serially diluted from 64,000 pM to 739.91 pM. Plasma samples (in triplicate) were diluted 1:10 in a 200 nM ANSA/DMSO solution in TBST. T3-HRP (SQRX-T3HRP.1, Squarix, Marl, Germany) was used as a competitor in the assay at a concentration of 15 nM in TBST (50 mM Tris, 150 mM NaCl, pH 7.6 and 0.1% Tween 20). Diluted samples (in triplicate) and standards (in quadruplicate) were added to the assay plates along with the T3-HRP and incubated at RT for 30–40 min. All reagents were accurately dispensed using an Epmotion 5075 liquid handling robot (Eppendorf, Macquarie Park, Australia), followed by four TBST washes between each procedural step. Product development was visualised with a commercially prepared 3,3′,5,5′-tetramethylbenzidine solution (TMB Core+, Bio-Rad Laboratories, Granville, Australia), arrested with 0.2 M sulphuric acid and measured by a Spectramax M3 plate reader (Bio-Strategy, Tullamarine, Australia) set at 450 nm.

### 2.4. Statistical Analysis of Physiological Parameters and Plasma Hormone Concentrations

Two animals in each cohort did not cope with the conditions and were removed from the experiment as per animal ethics protocols. The analysis was performed on the data collected on the 10 steers of each cohort that completed the CCR period. Daily means have been aggregated into periods to enhance statistical power.

A Generalised Linear Model (GLM) of Analysis of Variance was performed on all variables using the SAS statistical software (version 9.4, TS1M1). These include 5 physiological parameters (DMI, RumT, RR, PS and RecT) and 11 plasma hormone concentrations (insulin2glucose, insulin, leptin, leptin_log10, adiponectin, prolactin_log10, TSH_log10, T4, T3, T3_log10, and T3_T4_ratio). Three fixed effects were evaluated for each variable to determine their significance on the trait, including cohort difference (2 levels), room variations (4 levels) and period variations (3 or 4 levels, depending on the specific variable). For example, the GLM model for DMI is outlined as follows:

DMI = mean + cohort + room(cohort) + period + period(Cohort) + error

Two interaction terms were also incorporated into the model. These include room(cohort), which refers to the nested effect of a room within a cohort, and period(Cohort), which represents the nested effect of a period within a cohort. The model allowed for examination of the impacts of both across and within individual factors on each variable.

For all analyses, the GLM model utilized Cohort 1, Room 1, and PreChallenge as the comparison baseline and generated: (a) predicted least-square means for individual fixed factors, as well as, the interaction terms, (b) predicted least-square effects for individual levels of a fixed factor. In addition, it conducted pairwise comparisons for all levels of individual fixed factors and their interactions, allowing for the identification of the specific level(s) that contributed to the significant results.

## 3. Results

### 3.1. Responses to High Heat Load

#### 3.1.1. Physiology and Performance

**Figure 2:**
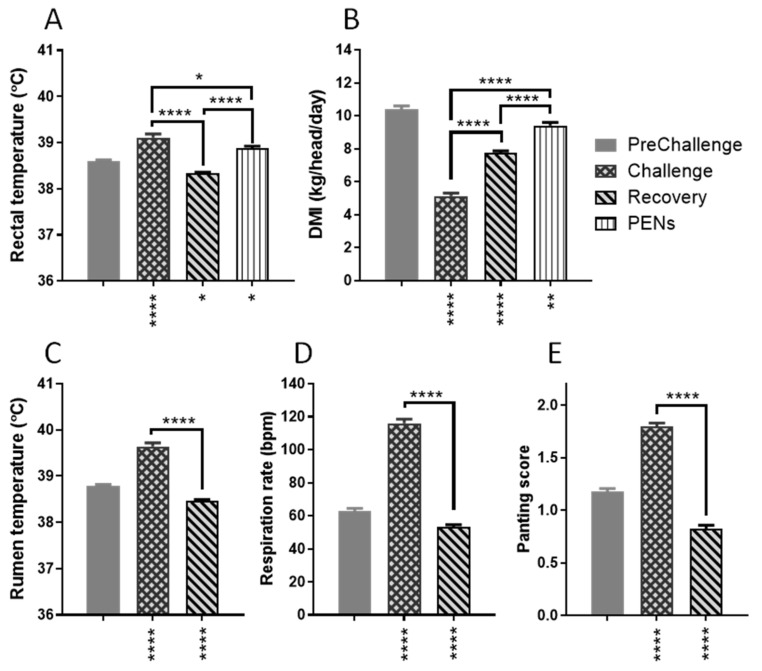
Animal physiological measures over the thermal challenge experiment. Each histogram presents the daily means ± SEM for each period. Panels (**A**,**B**), for rectal temperature and DMI during PreChallenge, Challenge, Recovery and PENS. Pen-level DMI, not individual DMI, was obtained during PENS. Panels (**C**–**E**), period means (±SEM) for rumen temperature, respiration rate and panting score during PreChallenge, Challenge and Recovery. Significant differences of subsequent periods with the PreChallenge mean are indicated by asterisks below the x-axis. Significant differences between the remaining period means are denoted asterisks above the relevant bracket. * *p* < 0.05; ** *p* < 0.01; **** *p* < 0.0001.

There were strong effects of period on rectal temperature (RecT), DMI, rumen temperature (RumT), respiration rate (RR) and panting score (PS) (*p* < 0.0001; Figure 2). As anticipated, the daily mean RecT during Challenge (mean ± SEM: 39.11 ± 0.08 °C) was significantly increased relative to all other periods. On average, the mean RecT during Challenge was 0.52 °C higher than the PreChallenge mean (38.59 ± 0.04 °C, *p* < 0.0001; Figure 2A). Of note, the Recovery mean RecT (38.33 ± 0.03 °C) was significantly lower than all other means; on average, it was 0.26 °C less than the PreChallenge mean RecT (*p* = 0.0381). In PENS, the mean RecT was 0.29 °C higher than that of PreChallenge (*p* = 0.0252; Figure 2A) despite cooling autumnal conditions (Figure 1). The daily mean DMI fell by approximately 50% during Challenge (5.12 ± 0.18 kg/head/day, *p* < 0.0001; Figure 2B). It improved during Recovery and PENS; however, the mean DMI in PENS (9.41 ± 0.19 kg/head/day) remained lower than the PreChallenge mean (10.39 ± 0.20 kg/head/day, *p* = 0.0036).

RumT, RR and PS were assessed in the CCR only, i.e., during PreChallenge, Challenge and Recovery. The daily mean RumT during Challenge (39.64 ± 0.08 °C) was higher (0.86 °C) than the PreChallenge mean (38.78 ± 0.04 °C, *p* < 0.0001; Figure 2C). The Recovery mean RumT (38.46 ± 0.03 °C) was significantly lower than the PreChallenge mean (0.32 °C, *p* < 0.0001). Daily mean RR during Challenge was approximately 2-fold higher than the PreChallenge mean (116.00 ± 2.47 bpm vs. 62.72 ± 1.72 bpm, *p* < 0.0001; Figure 2D). The Recovery mean RR (53.35 ± 1.29 bpm) was lower than the PreChallenge mean (*p* < 0.0001). Not unexpectedly, mean PS followed a similar pattern (Figure 2E). The mean PS during Challenge (1.80 ± 0.03) and Recovery (0.82 ± 0.03) differed from the PreChallenge mean PS (1.18 ± 0.03, *p* < 0.0001 in both cases).

**Figure 3:**
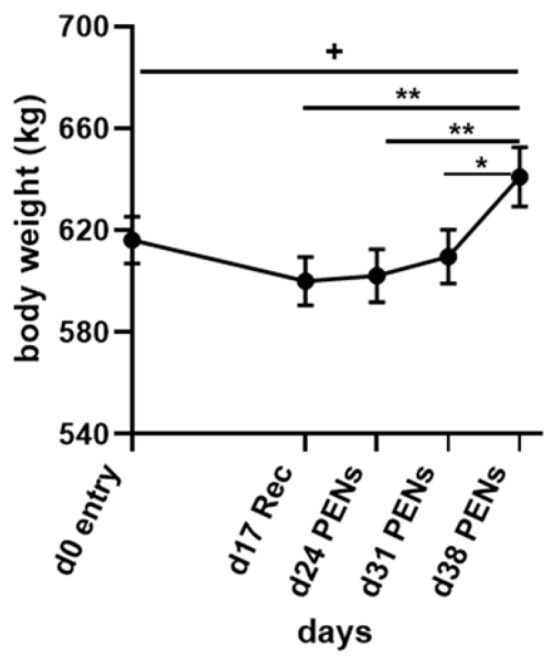
Mean body weights (±SEM) of the steers on entry to the CCR (day 0), day of exit (day 17) and three timepoints in PENS: days 24, 31 and 38. Significant differences amongst the means of each timepoint are indicated by the asterisks above the connecting bars. + *p* < 0.1; * *p* < 0.05; ** *p* < 0.01.

Body weights (BWs) were recorded on the day of entry into the CCR (day 0), the day of exit from the CCR (day 17) and days 24, 31 and 38 (during PENS) due to failure of the scales located in the CCR (Figure 3). The day 0 mean BW was 616.2 ± 9.23 kg. By day 17, on exit from the CCR, the mean BW had fallen to 600.00 ± 9.50 kg; however, this was not a significant reduction (*p* = 0.2626). The subsequent two BW measures on days 24 and 31 detected small increments that were not significantly different from day 0 or 17 mean BW (Figure 3). The BW gain on day 31, 14 days after exit from the CCR, was 9.6 kg/head. Only on day 38, when the mean BW rose to 641.1 ± 11.7 kg, was there a substantial increase in BW relative to days 17 and 24 (*p* ≤ 0.0099) and day 31 (*p* = 0.0358). There was a tendency towards a significant difference with day 0 mean BW (*p* = 0.0959).

#### 3.1.2. Pituitary Hormones—TSH and Prolactin (PRL)

**Figure 4:**
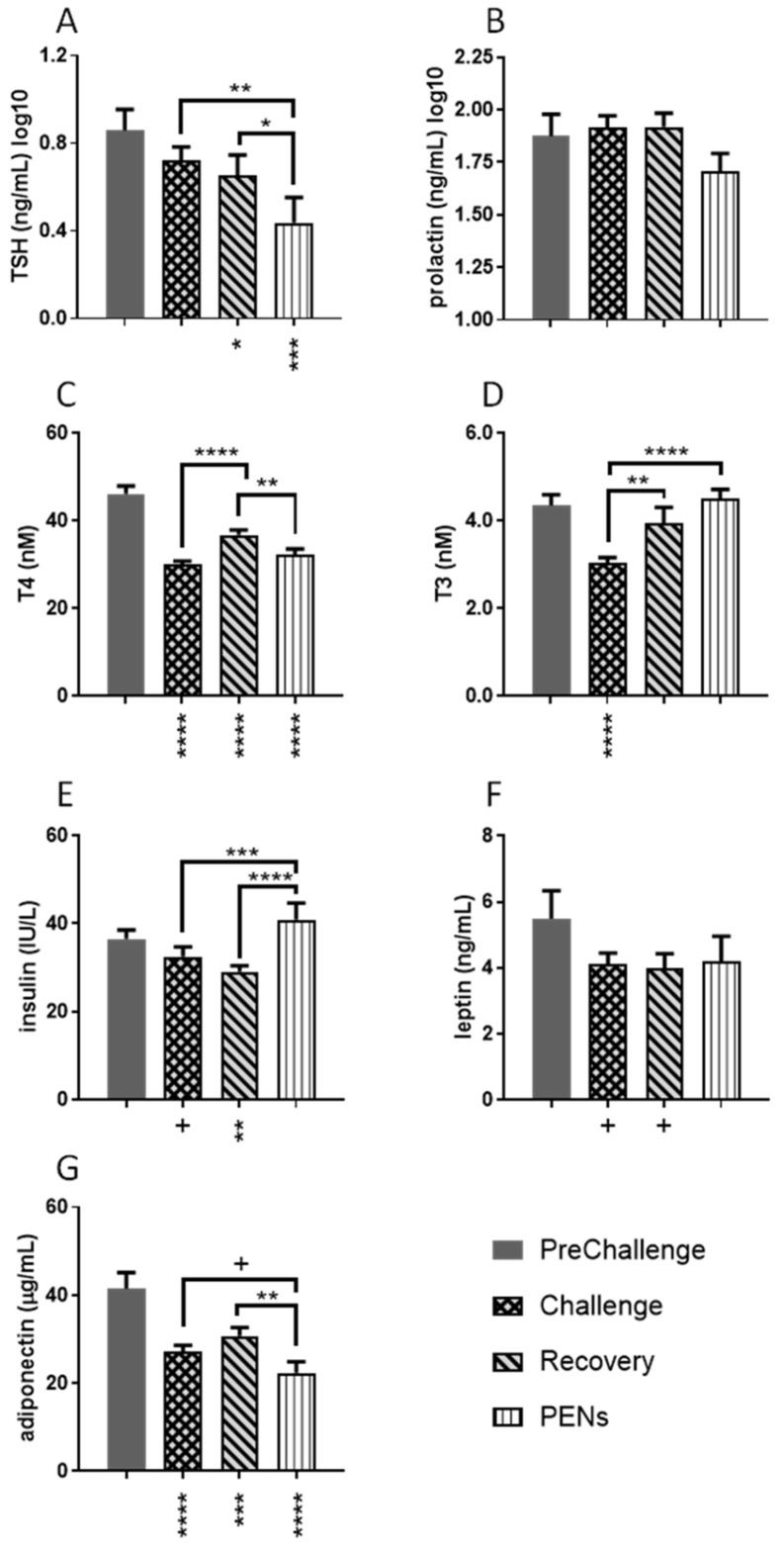
Plasma concentrations of the suite of hormones were measured over the four periods, PreChallenge, Challenge, Recovery and PENS. Each histogram presents the daily means ± SEM for each period (**A**) TSH log10, (**B**) prolactin log10, (**C**) T4, (**D**) T3, (**E**) insulin, (**F**) leptin and (**G**) adiponectin. Significant differences of subsequent periods with the PreChallenge mean are indicated by asterisks below the x-axis. Significant differences between the remaining period means are denoted by asterisks above the relevant bracket. + *p* < 0.1; * *p* < 0.05; ** *p* < 0.01; *** *p* < 0.001; **** *p* < 0.0001.

Due to large inter-individual variations in the plasma TSH and prolactin concentrations, the concentration data were log10-transformed for statistical analysis (Figure 4). There was an effect of period on mean log10 TSH levels (*p* = 0.0009; Figure 4A). The PENS mean was lower than the PreChallenge mean (*p* = 0.0001) and the Challenge mean (*p* = 0.0012). The Recovery mean was also less than the PreChallenge mean (*p* = 0.0397). There was no effect of period on mean log10-transformed PRL levels (*p* = 0.4423; Figure 4B), nor were there any differences amongst the period means.

#### 3.1.3. Thyroid Hormones—T3 and T4

There were strong effects of period on the mean concentrations of thyroid hormones, T3 and T4 (*p* < 0.0001 in both cases; Figure 4C,D). The mean T4 concentrations during Challenge, Recovery and PENS were significantly reduced compared to the PreChallenge mean (35, 20 and 30% lower, respectively, *p* < 0.0001 in all instances; Figure 4C). The Recovery mean T4 concentration (36.8 ± 1.12 nM) was greater than the PENS mean (32.2 ± 1.38 nM, *p* = 0.0058) and Challenge mean (30.1 ± 0.75 nM, *p* < 0.0001). The mean plasma T3 concentration during Challenge at 3.03 ± 0.13 nM was 30% less than the PreChallenge mean, and the lowest of all the period means (*p* ≤ 0.0013; Figure 4D). The means in Recovery and PENS were not different from the PreChallenge mean.

#### 3.1.4. Insulin, Leptin and Adiponectin

There was a strong effect of period on plasma insulin concentration (*p* = 0.0001; Figure 4E). The mean insulin concentration during Challenge (32.5 ± 1.14 IU/L) was reduced compared to the PENS mean (*p* = 0.0007) and tended to differ with the PreChallenge mean (*p* = 0.0897). In Recovery, the mean insulin concentration (29.1 ± 1.37 IU/L) was least of all the period means. It differed from the PreChallenge mean (36.5 ± 2.05 IU/L, *p* = 0.0056) and was reduced relative to the PENS mean (40.9 ± 3.83 IU/L, *p* < 0.0001; Figure 4E).

Leptin concentrations did not reveal any effect of period (*p* = 0.2347, Figure 4F). However, mean concentrations during Challenge and Recovery were ~25–27% reduced compared to the PreChallenge mean and tended to differ (*p* = 0.0584 and *p* = 0.0671, respectively; Figure 4F). In contrast to the leptin response, there was a strong effect of period on adiponectin concentrations (*p* < 0.0001; Figure 4G). Following PreChallenge, mean adiponectin concentrations were reduced compared to the PreChallenge mean (41.5 ± 3.61 µg/mL, *p* ≤ 0.0001; Figure 4G). The lowest concentration occurred in PENS (22.4 ± 2.48 µg/mL), which differed from the Recovery mean (30.7 ± 1.97 µg/mL, *p* = 0.0036) and tended to differ with the Challenge mean (*p* = 0.0617).

### 3.2. Relationships of Hormone Plasma Concentrations with THI and Rumen Temperature

**Figure 5:**
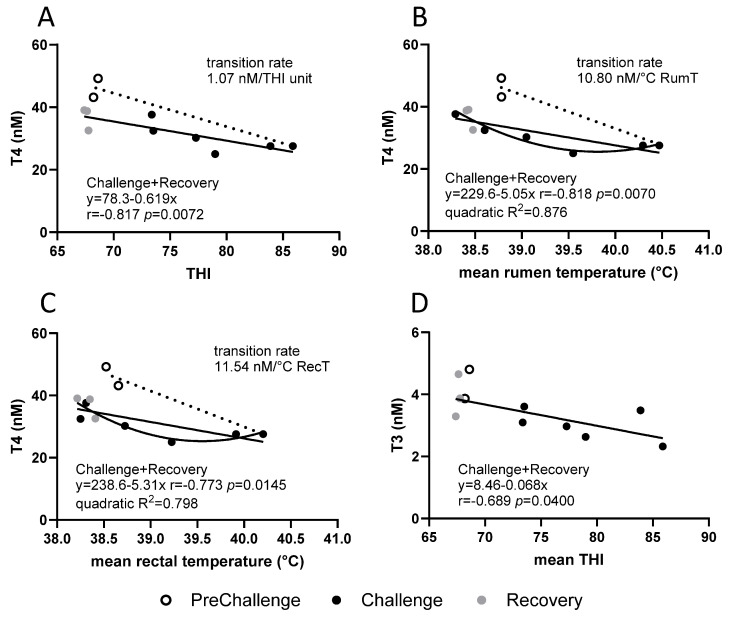
Relationships between daily mean plasma thyroid hormone concentrations and daily mean THI and core temperatures during the three periods in the CCR. (**A**) T4 vs. THI, (**B**) T4 vs. rumen temperature, (**C**) T4 vs. rectal temperature and (**D**) T3 vs. THI. The contribution from each period is indicated (see key). The line of best fit, linear equation, Pearson correlation r and significance level are provided. Quadratic models included for daily mean T4 concentration with core temperatures and the coefficient of determination, R^2^, are displayed. The dotted line represents the transition from PreChallenge to the first day of Challenge (day 7), and the rate of transition is given.

Under moderate heat load, the plasma concentrations of some of the hormones assayed here showed moderate to strong linear relationships with RumT and DMI during the interval in the CCR. From these relationships, the rates of change of the hormone concentrations per unit change in RumT and DMI were determined [17]. The analysis was applied to data from the current high-heat-load experiment to ascertain whether these relationships held. The daily mean concentrations of TSH, PRL, insulin, leptin and adiponectin did not exhibit obvious relationships with daily mean THI or core temperatures. Only T4 and T3 concentrations showed relationships with THI. T4 concentration was unique, displaying relationships with core temperatures (Figure 5).

An inspection of the plots (Figure 5A–C) showed that the PreChallenge T4 concentrations detracted from the linear relationships. The strongest relationship of mean T4 concentrations with daily mean THI occurred during the Challenge and Recovery periods (Pearson correlation r = −0.817, *p* = 0.0072; Figure 5A). Over this interval, T4 concentrations fell at a rate of 0.62 nM/unit THI. The transition rate from PreChallenge to the first day of Challenge (day 7) was more rapid at 1.07 nM/unit THI. The behaviours of T4 concentration with RumT and RecT were very similar to those with THI (Figure 5B,C). In both cases, the PreChallenge concentrations did not contribute to the linear relationships evident during Challenge and Recovery. Concerning daily mean RumT, a strong negative relationship was obvious (r = −0.818, *p* = 0.0070; Figure 5B). The linear equation indicated a rate of reduction of 5.05 nM T4/°C RumT. The transition rate from PreChallenge to the first day of Challenge (day 7) was almost double at 10.8 nM T4/°C RumT. However, a quadratic model was a good fit also (R^2^ = 0.876; Figure 5B). This behaviour suggests that under the conditions imposed on the first days of Challenge, T4 concentrations were reduced but stable over time when the daily mean RumT adhered to a range of 39.80–40.5 °C. As conditions cooled during the later days of Challenge and in Recovery, T4 concentrations rose when RumT fell below 39.85 °C (Figure 5B). As alluded to above, comparable behaviour was observed for the relationship between T4 concentration and RecT. There was a strong negative linear relationship during Challenge and Recovery (r = −0.773, *p* = 0.0145; Figure 5C) with a linear rate of change of 5.30 nM T4/°C RecT. The transition rate from PreChallenge to day 7 (first day of Challenge) was 11.54 T4/°C RecT. The polynomial model (R^2^ = 0.789) indicated that daily mean T4 concentrations rose as daily mean RecT fell below 39.70 °C (Figure 5C). The linear and quadratic relationships of T4 and RecT could be extended to PENS (r = −0.773, *p* = 0.0053; quadratic R^2^ = 0.721; Supplementary Figure S1A).

The relationships of daily mean T3 concentration with daily mean THI and core temperatures were not reminiscent of those of T4 concentration. Firstly, the moderately correlated negative linear relationship of T3 concentration with THI encompassed all periods (r = −0.689, *p* = 0.0400; Figure 5D). Thus, there was a consistent and bidirectional rate of change over the three periods at 68 pM T3/unit THI. Interestingly, when PENS data are included, the relationship has a higher correlation (r = −0.772, *p* = 0.0020; Supplementary Figure S1B) indicating a consistent T3 response over the 38 days of the experiment at 90 nM T3/unit THI. Secondly, no relationships were discernible for daily mean T3 concentrations and daily mean RumT or RecT.

### 3.3. Relationships of Hormone Plasma Concentrations with DMI

**Figure 6:**
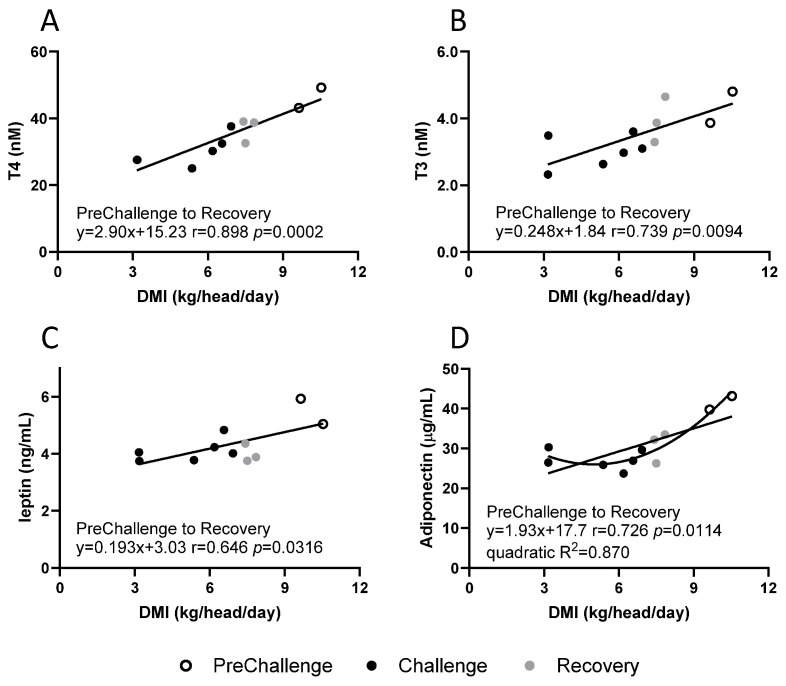
Relationships between daily mean plasma hormone concentrations and daily mean THI and core temperatures during the three periods in the CCR. (**A**) T4, (**B**) T3, (**C**) leptin and (**D**) adiponectin. The contribution from each period is indicated (see key). The line of best fit and the linear equation are shown, together with Pearson’s correlation r and the significance level. The quadratic model applied to the adiponectin relationship and the coefficient of determination, R^2^, are displayed.

No relationships were detected for daily mean concentrations of TSH, PRL and insulin with daily mean DMI. In the current experiment, mean daily DMI covered a broad range: 3.17–11.16 kg/head/day. Daily mean concentrations of T3, T4, leptin and adiponectin returned positive linear relationships with DMI (Figure 6). The T4 concentration exhibited a Pearson correlation r of 0.898 (*p* = 0.0002) with DMI, and the rate of change was 2.9 nM T4 per kg/head/day DMI (Figure 6A). The T3 concentration yielded a Pearson correlation r of 0.739 (*p* = 0.0094) with DMI (Figure 6B). In this case, the linear relationship with DMI extended to PENS (r = 0.796, *p* = 0.0011; Supplementary Figure S1C).

There was an overall positive but moderate linear relationship between daily mean leptin concentration and daily mean DMI (r = 0.646, *p* = 0.0316; Figure 6C) that predicted a rise of 0.19 ng/mL leptin per kg/head/day DMI. The relationship between daily mean adiponectin concentration and daily mean DMI appeared complex. A moderate linear relationship is evident (r = 0.726, *p* = 0.0114; Figure 6D), and a rate of change of 1.9 µg/mL per kg/head/day DMI was imputed. However, an inspection of the plot suggests that a quadratic relationship would be an appropriate fit (R^2^ = 0.870). This reflected the observation that at lower DMI (<7.0 kg/head/day), the daily mean adiponectin concentrations were maintained within the 23.7–30.3 µg/mL range.

## 4. Discussion

### 4.1. Physiological and Performance Responses

Overall, the physiological and performance responses of the steers in this study to thermal challenge were typical of most ruminants experiencing heat stress although the majority of the reports are from dairy cattle [1,2,20,21]. In Challenge, the steers were characterised by elevated mean core temperatures (RumT and RecT), a nearly 2-fold increase in mean RR and a 1.5-fold rise in mean PS, relative to thermoneutral conditions in PreChallenge. Daily mean DMI was significantly impacted by hyperthermia with a mean 50% decrease in intake during Challenge. It was apparent that there were larger changes in RumT between the periods than for RecT, implying that RumT was more sensitive to altered thermal conditions than RecT. However, these differences may be an artefact of measurement as the daily mean RumT for each steer was determined from the 1-hourly means in a 24 h interval as compared to RecT which was recorded about 7 a.m. daily, when core temperatures are most often at their minimum [22,23,24,25,26].

In Recovery, both measures of core temperature were significantly lower than the PreChallenge and Challenge means (0.26–0.32 °C) and indicative of a slight hypothermia or low normothermic state. This was despite improved DMI which remained ~25% lower than the PreChallenge DMI. In this hypothermic state, mean RR and PS were both significantly lower than PreChallenge means (15 and 30%, respectively). Mean LW at the end of the Recovery period (on exit from the CCR) showed no weight gain during Challenge and Recovery.

In PENS, DMI remained lower than the PreChallenge mean. RecT rose (0.29 °C greater than the PreChallenge mean) but fell short of the large increase in RecT in the moderate-heat-load (MHL) experiment where the difference was 0.57 °C. The gradual rise in RecT may indicate a lower metabolic rate in the high-heat-load (HHL) steers compared to the MHL counterparts. Indeed, the LW gain on exit from the CCR was slow. In the 14 days after exiting from the CCR, the steers in this experiment gained a mean of 9.6 kg/head in LW, with an overall linear rate of gain of 0.69 kg/head/day. By comparison, the steers subjected to MHL achieved a mean of 40.4 kg/head over the first 14 days in PENs, a linear rate of 2.90 kg/head/day. The differing responses suggest that compensatory growth was stalled or delayed in the HHL steers, supporting the concept of these steers having entered an allostatic state.

### 4.2. Pituitary Hormones—TSH and Prolactin

The secretion of TSH did not respond to the HHL Challenge but was reduced in Recovery and declined further in PENS. A similar pattern was observed in the MHL experiment, although there was no significant difference across the periods [17]. Kahl et al. (2015) [27] recorded substantial falls in plasma TSH in steers following 9 days of heat stress. Nevertheless, there are differing reports. Plasma TSH levels were impervious to 7 and 14 days of increased head load (35 °C maximum daily TA) in calves, yearling cattle and bulls (500–600 kg body weight) [28]. Similarly, Weitzel et al. (2017) [29] reported plasma TSH unaffected by heat stress in late-gestation and early-lactation Holstein cows. Detection of an effect of heat load on TSH secretion may be dependent on the heat load intensity and time of blood collection.

Unlike many reports of rising plasma prolactin concentrations in ruminants during heat stress, we could not detect a prolactin response. Generally, there appears to be a strong positive association between rising plasma prolactin concentrations and rising core temperature and/or TA in humans [30,31,32] and ruminants [28,33,34,35,36,37,38]. A review of the literature suggests that a greater than 1 °C in core temperature may be required to induce marked increases in the secretion of prolactin into the circulation. For example, Schams et al. (1980) [28] recorded 2-5-fold rises in prolactin concentration in heat-stressed calves, heifers and bulls, compared to concentrations obtained in thermoneutral conditions. In lactating cows, a 10-fold rise was achieved with four days of heat stress [39]. In the current study, the daily mean RecT during Challenge was 0.53 °C higher than the PreChallenge mean; however, the mean RecT and RumT for days 7 and 8 (days 2 and 3 of Challenge) were 1.3–1.7 °C higher, respectively, than the PreChallenge means. There was no spike in plasma prolactin concentration on those days.

### 4.3. Metabolic Hormones

**Figure 7:**
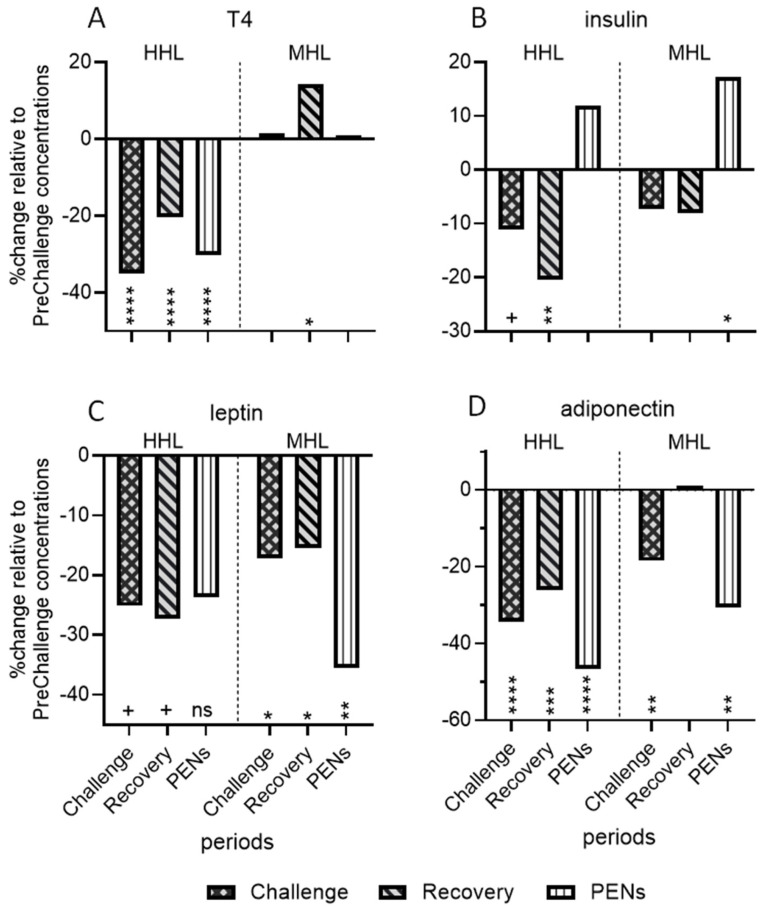
Comparison of the hormone response over the Challenge, Recovery and PENS periods relative to PreChallenge in the current high-heat-load (HHL) experiment and the earlier moderate-heat-load (MHL) experiment (Wijffels et al., 2023) [17]. (**A**) T4, (**B**) insulin, (**C**) leptin and (**D**) adiponectin. The period means are presented as % change from the PreChallenge mean in each case. The level of significant difference from the PreChallenge mean is denoted by the asterisk(s) above the x-axis. ns, not significant; + *p* < 0.1; * *p* < 0.05; ** *p* < 0.01; *** *p* < 0.001; **** *p* < 0.0001.

Figure 7 has been produced to aid the comparison of the hormonal responses in the moderate- and high-heat-load experiments. This figure shows the % changes from PreChallenge levels for T4, insulin, leptin and adiponectin (T3 was not measured in the MHL experiment).

#### 4.3.1. Thyroid Hormones—T3 and T4

The HHL regime employed in this study impacted both plasma T3 and T4 concentrations with differing behaviours across the four periods. In the three periods following PreChallenge, the mean plasma T4 concentrations were markedly depressed. There was a substantial fall in Challenge (~35%), a rebound in Recovery, although the concentrations were still 20% reduced from PreChallenge levels, and a subsequent significant reduction in PENS (Figure 7A). The overt depression of T4 concentrations was not evident in the MHL experiment where there was no decline in plasma T4 concentration in Challenge, a rise of ~10% in Recovery and a return to PreChallenge levels in PENS (Figure 7A; Wijffels et al., 2023) [17]. Plasma T3 concentrations in the HHL Challenge also declined about 30% relative to PreChallenge but returned to PreChallenge levels in Recovery and PENS.

Johnson and Ragsdale (1960) [40] demonstrated reduced thyroid activity (^131^I release rates) in heat-stressed heifers, which explains the reduced levels of circulating thyroid hormones during Challenge in the current study and many other studies of thermal challenge in ruminants [27,29,41,42,43,44,45]. Only McGuire et al. (1991) [46], working with lactating cows, found higher T3 and T4 concentrations during thermal challenge compared to the concentrations in feed-restricted thermoneutral (FRTN) cows.

Highlighting the complexity of the regulation of circulating levels of T3 and T4, there is no clear relationship between the concentrations of these two hormones during thermal challenge. For example, Mohammed and Johnson (1985) [36] reported reduced T3 levels but unchanged T4 levels with thermal stress of lactating cows compared to concentrations obtained in thermoneutral conditions. Greater reductions in plasma T3 concentration during heat stress compared to the fall in T4 concentration have been reported for lactating cows and heifers [42,45]. In nine-day heat-stressed steers, Kahl et al. (2015) [27] found the reverse, with a greater reduction in T4 concentration than that for T3 when compared to pair-fed thermoneutral (PFTN) counterparts. Weitzel et al. (2017) [29], working with cows in late pregnancy or early lactation, found that both hormones fell by similar proportions when the cows were subjected to heat stress and compared to PFTN cows.

In Challenge, the unchanged plasma TSH concentration alongside reduced circulating concentrations of both T3 and T4 is typical of a thyroid Type 1 allostatic response [47]. The inability to meet the energy demand to respond to a stressor sets up a Type 1 allostatic load. One of the consequences for the thyroid hormones is reduced conversion of T4 to T3 (hypodeiodination) due to the downregulation of peripheral (tissue) deiodinases 1 and 2 (DIO1 and 2) [47,48]. The expression of DIO3, which encodes another peripheral DIO responsible for the inactivation of both T3 and T4, is upregulated in many conditions that set up a Type 1 allostatic load [47,48,49].

The expression of DIOs appears to be impacted by thermal challenge. Pigs after eight days of heat stress exhibited a ~60% reduction in hepatic DIO1 enzyme activity relative to PFTN pigs [50]; however, Kahl et al. (2015) [27] found no differences in hepatic DIO1 enzyme activity between heat-stressed and thermoneutral steers. Weitzel et al. (2017) [29] reported a large fall in hepatic DIO1 transcription in early-lactation cows relative to both thermoneutral conditions and the PFTN counterparts, but not in the late-pregnant animal. Similarly, heat-stressed broiler chickens showed a 2-fold reduction in hepatic DIO1 transcription and a 3-fold increase in hepatic DIO3 transcription [51].

Following recovery from HHL challenge, the steers initiated high rates of weight gain only after day 30 (at the earliest). As the experiment did not extend beyond day 38, the full trajectory of compensatory gain was not captured. We know that rises in plasma T3 and T4 concentrations can be gradual during refeeding after short-term fasting in cattle [52] and longer-term feed restriction [53,54,55]. In the current study, plasma T4 levels remained lower than baseline right through to PENS, suggesting that thyroid output was still limited. The low plasma TSH concentrations in Recovery and PENS are likely to have influenced T4 production and secretion. Plasma T3 levels, on the other hand, had recovered rapidly, suggesting a role for the peripheral DIOs.

In exploring the relationship between T4 concentration and THI, it became evident that both PreChallenge and PENS were different states and not contiguous with the change in thyroid T4 output (and modulation of DIO activities) as the steers adjusted to the new conditions in Challenge and Recovery. There was a rapid transition in T4 concentration from PreChallenge to a much-reduced concentration on day 7 (the first day of Challenge). Subsequently, there was a gradual linear rise in T4 concentration through Challenge and Recovery which was 60% slower than the transition rate. The relationships of T4 concentration with daily mean RumT and RecT were not dissimilar to that of daily mean THI. There was a rapid reduction in T4 concentration as core temperatures achieved their maxima on day 7. This was followed by a gradual increment in T4 concentration as core temperatures fell to the slightly hypothermic state observed in Recovery. The rate of rise in T4 concentration during Challenge, Recovery and PENS, in the case of RecT, was about half that of the transition rate (46–47%). However, the incremental rise in T4 concentration relative to the core temperatures during Challenge and Recovery appeared to be non-linear (e.g., quadratic) in the MHL experiment where there was a constant rate of change in T4 concentration of 9.7 nM T4/°C RumT across all three periods [17]. Interestingly, this rate was very similar to the transition rate in the current HHL experiment during Challenge and Recovery. Is there a limit as to how quickly the thyroid can reduce its synthesis and/or secretion of T4 considering that plasma TSH is not changed in Challenge? Or is the fall in plasma T4 entirely due to the action of the DIOs?

For the HHL experiment, there was a positive linear relationship with DMI throughout PreChallenge to Recovery with a predicted rise of 2.90 nM T4 per kg/head/day. T4 concentrations appeared to be highly responsive to DMI. The relationship between T4 concentration and DMI was the strongest of all those pertaining to T4 concentration. On the other hand, in the MHL experiment, no relationship between T4 concentrations and DMI was detected as DMI displayed an elliptical relationship with RumT [16,17].

The behaviour of the daily mean T3 concentrations at the level of the period means differed from that of T4. The distinctive T3 response was further evident with an inspection of the relationships between T3 concentrations and THI, RumT and DMI. Other factors influence the plasma T3 concentration during thermal challenge and recovery. T3 concentration displayed a strongly correlated linear relationship with THI across all periods; there was no rapid transition to Challenge, and the PENS data contributed to the relationship. However, there was no discernible relationship with RumT. Interestingly, Hunninck et al. (2020) [56] found air temperature to be the best predictor of faecal T3 levels in wild impalas and a superior predictor of feed intake. In the HHL experiment, T3 concentration possessed a highly correlated linear relationship with DMI across all periods, including PENS. Kong et al. (2004) [57] demonstrated that T3 directly acts within the hypothalamus to regulate feed intake.

#### 4.3.2. Insulin

Despite the 50% reduction in DMI during Challenge, plasma insulin was not significantly affected. When most animals undergo a sudden reduction in feed intake and consequent fall in blood glucose, the production and secretion of insulin are reduced, and plasma insulin levels fall. This is typically observed with short-term fasting or underfeeding of ruminants also [52,58,59]. However, insulin responses in ruminants subjected to various heat loads in climate-controlled chambers have been broad and varied. Studies on lactating and non-lactating cows, heifers, calves and ewes have returned very inconsistent responses with increases, decreases or no change whether compared to baseline levels (pre-challenge) or plasma insulin in ad lib or PFTN counterparts [39,60,61,62,63,64,65,66,67,68,69].

In the current investigation, the overall pattern of changes in insulin concentration relative to PreChallenge levels was reminiscent of that seen in the MHL experiment (Figure 7B). A major difference between the two thermal challenge experiments was a tendency toward a significant reduction in insulin concentration during Challenge, followed by a marked fall in Recovery in the HHL experiment. This coincided with a partial return of feed intake and lowered core temperatures. It is possible that the latter influenced insulin concentration since hypothermia is known to suppress insulin secretion [70,71,72]. In the MHL experiment, the animals did not experience lowered core temperatures in Recovery [16,17]. Another difference between the two thermal challenge experiments was the absence of a strong rise in insulin concentration in PENS in the HHL experiment. In the MHL experiment, insulin concentrations were on an upward trajectory after Recovery in response to rising blood glucose concentrations and setting up insulin resistance in PENS [17]. Insulin concentration showed no discernible relationship with climatic conditions, core temperatures or DMI in both experiments.

#### 4.3.3. The Adipokines

Leptin, a protein secreted by adipose tissue, contributes to appetite regulation, availability of energy substrates (reviewed in [73,74]) and signalling to the hypothalamus as to the extent of the body’s fat stores (reviewed in [75,76]). Leptin acts as a brake on feed intake. Fasted or underfed ruminants experience falling levels of plasma leptin which encourages feed intake [75,77,78,79,80]. Heat-stressed steers under the MHL regime managed falls in leptin in Challenge and Recovery (Figure 7C), although the decrease was less than that experienced by the FRTN counterparts [17]. By comparison, only a tendency toward significant falls in circulating leptin concentrations during Challenge and Recovery was detected in the HHL experiment so that the suppressive effect of leptin on appetite was weakly modulated. Importantly, leptin concentrations did not fall significantly in PENS; thus, this neuroendocrine signal to increase DMI did not occur. Figure 6C shows that leptin concentration was at a minimum when DMI was most reduced (days 6–8), so patently, there was a leptin response, at least initially. Despite the different responses in the two thermal challenge experiments, leptin concentration returned moderate positive linear relationships with DMI in both cases [17]. Also consistent was that leptin concentration did not appear to be associated with core temperature in both experiments.

A clear contrast with the MHL experiment was the lack of response by leptin in PENS (Figure 7C). Leptin concentrations were 35% less in the MHL experiment, contributing to DMI returning to PreChallenge levels and fuelling compensatory growth (Figure 7C). In the HHL experiment, circulating leptin concentrations were not significantly reduced in PENS and therefore did not participate in promoting feed intake. This may have contributed to the limited recovery of DMI and thus live weight gain in the HHL experiment.

Adiponectin is an adipokine also, but unlike leptin, its circulating concentration is negatively correlated with adiposity and does not reflect short-term changes to feed intake (reviewed in [81]). The adiponectin response to Challenge in the current experiment was distinctive, with an immediate and sustained fall (~35%) in plasma concentration. Reduced adiponectin levels persisted throughout the next two periods. This pattern differed from the MHL experiment where adiponectin concentrations returned to PreChallenge levels in Recovery (Figure 7D). Furthermore, the %change (reduction) in adiponectin concentration in Challenge and PENS in the MHL experiment was less than that of the HHL experiment.

While adiponectin concentrations in thermoneutral underfed animals are not altered, moderately to strongly correlated negative linear relationships with DMI are evident in the MHL and HHL experiments (ref. [17] and this paper). DMI fell to 3 kg/head/day in the HHL experiment, and a quadratic relationship better described the interaction of adiponectin concentration and DMI. This is suggestive that as DMI drops below a certain threshold, in this case, ~7.3 kg/head/day, adiponectin secretion although much reduced, stabilised. In the MHL experiment, the DMI of the thermally challenged steers ranged over 7–11 kg/head/day and delivered a strongly correlated negative linear relationship [17]. The altered behaviour of adiponectin concentration in the HHL experiment relative to the MHL experiment was the absence of a relationship between adiponectin concentration and core temperature(s).

### 4.4. The Endocrine Milieu During and After the High-Heat-Load Challenge

Different patterns of hormone responses occurred in the HHL experiment. The dominant hormone responses can be ascribed to marked and persistent declines in the plasma concentrations of T4 and adiponectin, and possibly low leptin levels, from Challenge through to PENS. Insulin levels were depressed also during Challenge and Recovery (but not PENS), and TSH levels during Recovery and PENS. The new endocrine state aligns with the concept that these steers produced an allostatic response to the HHL challenge as indicated by the stably reduced core temperatures, RR, DMI and PS that occurred in Recovery and PENS.

What are the metabolic consequences of enduring depressed levels of circulating T4 and adiponectin in what should be a rapidly growing animal? And how does the overlay of reduced insulin and leptin for two of the periods influence the metabolism of these steers? It is difficult to construct an understanding of the metabolic consequences of such a hormone milieu. The action of these hormones within the same tissue can be contradictory. Additionally, there is limited detailed study of the regulatory dynamics and interactions between the hormones (and their numerous co-regulators) in ruminants, so we are reliant on numerous intensive studies of rodent models, explants or cell lines, and some input from mostly human clinical studies [82]. However, it may be helpful to note that under the conditions of the HHL experiment, there were two distinct groupings of the hormones. There was a strong positive linear relationship between adiponectin and T4 concentrations during PreChallenge to Recovery (r = 0.885, *p* = 0.0003; Supplementary Figure S2A). This observation concurs with a report of a strong correlation between the serum concentrations of T4 and adiponectin in euthyroid, hyperthyroid and hypothyroid rats [83,84]. Leptin and insulin concentrations correlated positively over the same three periods (r = 0.801, *p* = 0.0030; Supplementary Figure S2B). Leptin appears to exert influence over insulin’s activities in many tissues, and insulin stimulates the synthesis of leptin (reviewed in [85]).

The impact of the hormone milieu on the liver suggests that the lower availability of the thyroid hormones should suppress their promotion of hepatic gluconeogenesis, fatty acid (FA) uptake and oxidation and de novo lipogenesis (reviewed in [82,86]) as well as cholesterol synthesis and efflux (reviewed in [87,88]). On the other hand, the lower adiponectin levels would be predicted to encourage hepatic lipogenesis and gluconeogenesis [74,89]. Similar to the low thyroid hormones, the reduced adiponectin levels would limit this hormone’s ability to enhance hepatic FFA oxidation. The low levels of leptin would endorse lipogenesis but suppress FA oxidation [74]. On the other hand, reduced insulin levels would limit hepatic lipogenesis but reduce its constraint on FA oxidation and gluconeogenesis in the liver [89]. The counteractive influences of these hormones make it difficult to arrive at an overall picture of hepatic energy metabolism during and after the HHL challenge and recovery.

The scenarios for skeletal muscle and adipose tissue seem less complex. There was likely an overall suppression of energy metabolism and reduced insulin sensitivity. In skeletal muscle, the lowered concentration of thyroid hormones would dampen mitochondrial oxidation, insulin sensitivity and consumption of glucose [87]. Notably, skeletal muscle expresses DIO2 which converts T4 to T3 locally [82]. The low adiponectin levels would also reduce insulin sensitivity and glucose uptake, along with diminishing FA oxidation [74,89]. The decreased leptin concentrations would further discourage FA oxidation, glucose uptake, insulin sensitivity and muscle growth [74,76]. Insulin’s well-known roles in promoting glucose uptake and consumption, and inhibition of FA oxidation in muscle and gluconeogenesis, would be limited by its lower concentrations [73,89]. Likewise, insulin’s action of maintaining or contributing to muscle growth by encouraging protein synthesis and depressing protein degradation would be hampered [90]. It would appear overall that the energy metabolism in skeletal muscle was discouraged regardless of the energy substrate. It is also likely that muscle growth (protein deposition) is constrained. The skeletal muscle is not receiving endocrine signalling that promotes an anabolic state, but it is not clear that the muscle is in a catabolic state.

Generally, each of the hormones has similar effects on adipose as on skeletal muscle albeit with some adipose-specific functions. Lowered thyroid hormone levels will depress the stimulation of lipolysis with the consequent suppression of the release of FA into circulation [86,88]. The lower levels of adiponectin would dampen expansion in adipose depots, resulting in less lipid storage, but on the other hand, may still be permissive of lipolysis [74,91]. Counteractive to this, low levels of leptin may even enhance glucose uptake in adipose [76] but reduce lipolysis [74]. Largely, there was weakened signalling for fat deposition, but the role of adipose in the provision of FA was not clear during the HHL experiment.

Besides the overall changes in endocrine status as a consequence of HHL, we were interested in seeing if the interactions of the hormone concentrations with THI, core temperatures and DMI differed from those detected in the MHL experiment. The positive relationships of the two adipokines with DMI were conserved in both thermal challenges. However, adiponectin’s positive correlation with RumT in the MHL experiment was not reproduced by HHL. The interaction of T4 concentration with core temperatures was maintained regardless of heat load, but relationships with DMI and THI were apparent in HHL only. These findings show that generalisations about endocrine responses to heat load without reference to the context should be avoided.

### 4.5. Limitations of the Study

Amongst the limitations of this study to be acknowledged are that the steers were maintained in thermoneutral conditions during PreChallenge and Recovery. Barring the occurrence of a highly unseasonal heatwave, most cattle will experience strong heatwave conditions after some level of summer season ‘adaptation’. Thus, the responses in these animals may differ from the steers in this study. Additionally, PFTN or FRTN treatment was not conducted. The anticipated reduction in DMI that would have been imposed on 500–600 kg steers with no prior experience of extreme reductions of food intake and being housed in a closed environment would have induced a stress response. There were indications of confinement or overall housing and management stress in the FRTN group in the MHL experiment [17].

The PENS period had only two scheduled bleeds. In hindsight, more frequent bleeds during the PENS phase would have yielded important information on the hormone responses during this phase and improved understanding and interpretation of the depth and persistence of putative allostatic response. This phase is clearly grounds for future research. The findings of this paper and the associated physiology paper [13] point to parameters that could inform genetic selection within the Angus breed at least. While there are potential ‘markers’ for heat tolerance or resilience (e.g., the relationship between T3 concentration and THI, or T4 concentration and DMI), robust genetic studies require the phenotyping of thousands of animals. The data supplied by this experiment were limited to two cohorts of 10 Black Angus steers.

## 5. Conclusions

The intensity (and most likely duration) of heat load will alter how the endocrine system responds to the environmental heat load and the physiological responses to it. The comparison of a moderate heat load and a high heat load indicates that differing levels of stress elicit differing physiological and hormonal strains. Based on their physiological responses, we speculated that the HHL steers have undergone an allostatic response [13]. The culmination of the study of their metabolic hormone responses revealed that the HHL challenge and recovery from it produced an endocrine milieu that metabolically constrained the animal after the heat load event, a situation that supports the concept of allostasis in these steers. It is possible that as a consequence of HHL, the steers have attained new set points to ensure reduced feed intake and growth rates that lessen endogenous heat production while conditions threatened another potentially life-limiting event.

Ideally, in hot environments, production animals should be restricted to their homeorhetic core temperature range. This would enable the animals to adjust their physiology, endocrine and metabolic states appropriately and allow their facile re-adjustment as thermocomfortable conditions are reinstated. Inevitably, strong heatwaves that force production animals, especially feedlot animals, out of their homeorhetic range will occur. The use of thorough production and management records by feedlot managers and allied professionals will assist in identifying the conditions or events for their site that engender a homeorhetic response or an allostatic response for the various differing cohorts of cattle (e.g., short-fed, long-fed, days-on-feed, breed, etc.).

Two major approaches can be applied to ameliorate heat stress in dairy and feedlot cattle: the adjustment of the environment to avoid the accumulation of heat load and the adjustment of feed rations to reduce rumen and enteric heat production (and consequently hepatic heat production). These concepts and their practical application have been reviewed and revisited over the decades [92,93,94]. While there has been solid progress in the former [8,9,10,11,12], there has been less success in modifying rations to ameliorate heat stress. In part, the rapid reduction in feed intake and its persistence in recovery confound the delivery of supplements that may have benefits. Perhaps the simplest approach is to reduce the amount of feed on offer or the metabolisable energy of the ration in the days prior to a strong heatwave. However, this requires that the feedlot managers and their advisors have high levels of confidence in the prediction of heat wave onset and intensity at their facility.

## Data Availability

Files (xlxs) containing the physiological and endocrine data can be obtained from the corresponding author.

## Supplementary Materials

The following supporting information can be downloaded at https://www.mdpi.com/article/10.3390/ani15020251/s1: Figure S1: Relationships between daily mean plasma thyroid hormone concentrations and core temperatures, THI or DMI, from PreChallenge to PENS; Figure S2: Relationships between the hormones over PreChallenge to Recovery; Table S1: Assay performance (mean ± SEM). Reference [95] is cited in the supplementary materials.
