# Supplementary material for: Stress and Strain: Differentiating the Responses to High and Moderate Heat Loads and Subsequent Recovery in Grain-Fed Feedlot Steers—Metabolic Hormones
Source: Animals (Basel). 2025 Jan 17;15(2):251. doi: 10.3390/ani15020251 (PMC11758642; doi:10.3390/ani15020251)
Supplement: Supplementary file 1 [file animals-15-00251-s001.zip › animals-3380270-supplementary.pdf]

Supplementary Table 1. Assay performance (mean ± SEM). The limits of the blank and detection were calculated according to [95].

Analyte	Limit of the Blank	Limit of Detection	Intra-assay %CV	Inter-assay %CV
Prolactin (ng/mL)	10.7±2.0	15.3±1.9	2.8±0.1	11.6±1.6
Adiponectin (μg/mL)	1.2±0.2	1.5±0.2	2.9±0.13	11.1±1.1
Leptin (ng/mL)	0.4±0.1	0.6±0.1	2.5±0.20	9.8±1.8
TSH (ng/mL)	0.7±0.0	0.8±0.0	2.6±0.10	6.0±0.70
T4 (nM)	0.1±0.0	0.2±0.0	3.5±0.6	13.2±1.6
T3 (pM)	482.4±63.8	1031.8±64.3	11.6 ± 1.8	14.1± 1.1

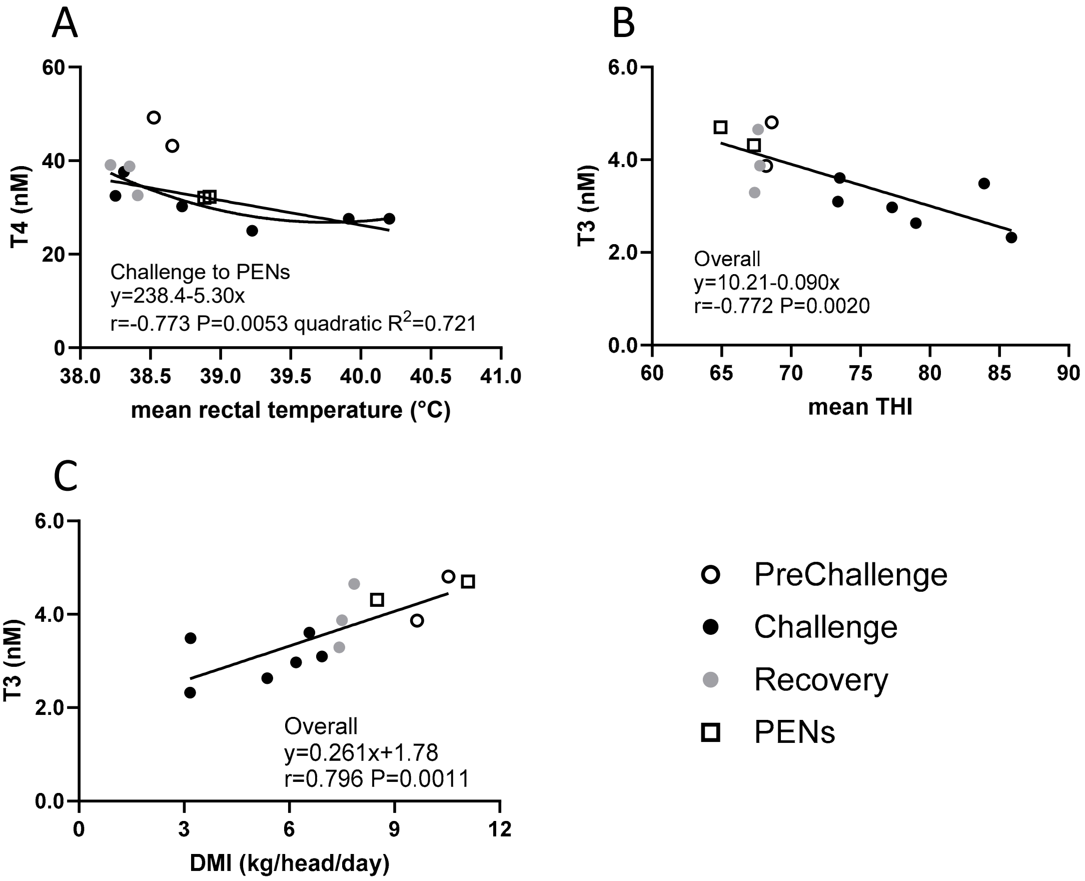

Supplementary Figure 1. Relationships between daily mean plasma thyroid hormone concentrations and core temperatures, THI or DMI, from PreChallenge to PENs. (A) daily mean T4 concentrations vs. daily mean rectal temperature from Challenge to PENs; (B) daily mean T3 concentrations vs. daily mean THI for the entirety of the experiment (PreChallenge to PENs); (C) daily mean T3 concentrations vs. daily mean DMI from PreChallenge to PENs. The contribution from each period is indicated (see key). The line-of-best fit and linear equation are given along with the Pearson correlation r and the level of significance. The quadratic model applied to the T4 relationship and the coefficient of determination, R^2, is displayed.

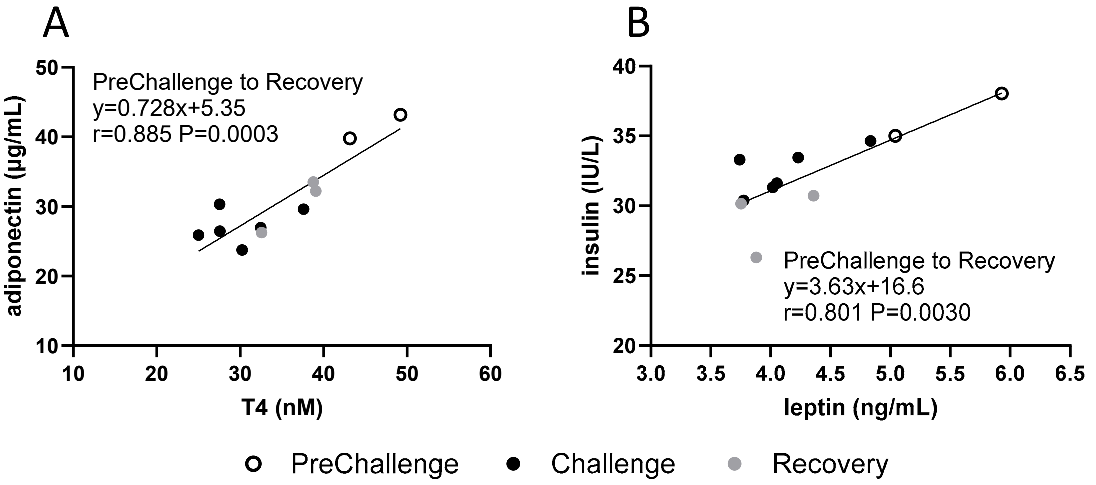

Supplementary Figure 2. Relationships between the hormones over PreChallenge to Recovery. (A) daily mean concentration of adiponectin vs T4; (B) daily mean activity of insulin vs daily mean leptin concentration. The contribution from each period is indicated (see key). The line-of-best fit and linear equation are given along with the Pearson correlation r and the level of significance.
